# Preparation and Characterization of Ternary Antimicrobial Films of β-Cyclodextrin/Allyl Isothiocyanate/Polylactic Acid for the Enhancement of Long-Term Controlled Release

**DOI:** 10.3390/ma10101210

**Published:** 2017-10-20

**Authors:** Jinpeng Wang, Chao Qiu, Ganesan Narsimhan, Zhengyu Jin

**Affiliations:** 1 State Key Laboratory of Food Science and Technology, Jiangnan University, Wuxi 214122, China; q930017357@163.com (C.Q.); fpcenter@jiangnan.edu.cn (Z.J.); 2 School of Food Science and Technology, Jiangnan University, Wuxi 214122, China; 3 Synergetic Innovation Center of Food Safety and Nutrition, Jiangnan University, Wuxi 214122, China; 4 Department of Agricultural and Biological Engineering, 225 South University Street, Purdue University, West Lafayette, IN 47907, USA; narsimha@purdue.edu; 5 International Joint Laboratory on Food Safety, Jiangnan University, Wuxi 214122, China

**Keywords:** allyl isothiocyanate, inclusion complex, antimicrobial activities, microstructure, controlled release

## Abstract

Allyl isothiocyanate (AITC) are natural essential oil components that have outstanding antimicrobial activities. However, low water solubility, high volatility, and easy degradation by heat, restricting their application in food packing industry. Development of the inclusion complex of β-cyclodextrin/AITC (β-CD/AITC) is a promising solution. Furthermore, the incorporation of β-CD/AITC complex into polylactic acid (PLA) films would be an attractive method to develop food antimicrobial materials. The aim of this study was to evaluate the enhancement in physicochemical properties, antimicrobial activities, and controlled release of β-CD/AITC from such films. The addition of β-CD/AITC significantly increased the flexibility and thermal stability of films. The Fourier transform infrared (FTIR) results revealed that the interactions between β-CD/AITC and PLA films occurred. The controlled release of AITC encapsulated in β-CD was significantly affected by relative humidity and temperature. The PLA films containing β-CD/AITC can be applied as an effective antimicrobial packing material for food and non-food applications.

## 1. Introduction

Allyl isothiocyanate (AITC), a natural antimicrobial essential oil component with a volatile and pungent character, has gained considerable attention because of its important applications as a naturally derived preservative [1,2] and in food packaging applications [3,4]. Natural preservatives are perceived to have lower toxicity, less negative environmental effects, and a better consumer acceptance [5]. AITC is a plant derived antimicrobial agent that exhibits outstanding antimicrobial characteristics that may reduce the conventional use of synthetic food preservatives by replacing them with natural antimicrobial compound [6]. As for making active packaging materials, AITC used alone or in combination with modified atmosphere packaging has demonstrated effectiveness in inhibiting bacterial growth in solid food models, cheeses, and chicken meat [7,8,9,10]. However, the following points limit their widespread use as packaging materials, both for food or non-food applications. First, AITC is poor water solubility [11] owing to a high hydrophobicity and that it may limit contact with pathogens in high moisture content foods or materials. Second, its pungency makes its direct incorporation into food matrices undesirable [12]. Third, AITC exhibits thermal instability during food or packaging materials processing. Therefore, the AITC needs to be encapsulated in a hydrophilic shell material in order to improve its solubility, improve its organoleptic properties, control its release rate, thereby prolonging its effectiveness [13]. Cyclodextrins (CDs) are a group of natural cyclic oligosaccharides, which are formed as a result of enzymatic degradation of starch or starch derivates [14]. The common forms are α-, β-, and γ-CDs composed of 6, 7, and 8 α-1, 4-linked glucose units, respectively [15]. Owing to their hydrophobic cavity, CDs are able to form host–guest complexes via weak forces, such as van der Waals interactions, dipole-dipole interactions, and hydrogen bonding. The inclusion complex (IC) of CD with a guest molecule has advantages, such as higher solubility, higher thermal stability, and bioavailability of hydrophobic guests; which assists in the controlling of volatility, masking of unpleasant odors, and controlling the release of drugs and flavors [10,13]. In recent years, some studies have reported the preparation of the IC of CD for the encapsulation of AITC. Li et al. reported that AITC was encapsulated by a α- and β-CD using a coprecipitation method [3]. Polylactic acid (PLA) is a biomass-oriented polyester that can be synthesized either from lactic acid or by ring-opening polymerization from its dimer, lactide. The monomer, lactic acid, obtained from the fermentation of corn starch, is also the final degradation product of PLA, and is considered as nontoxic and environmentally benign [16,17]. Because of these advantages, PLA has been extensively studied for food packaging applications [18,19,20,21]. However, PLA’s practical applications are often limited by its inherent brittle nature and low thermal stability [22]. There is limited literature on the applications of AITC loaded β-CD in the PLA films. Plackett et al. reported that the thermal stability of AITC encapsulated in α-CD or β-CD in polylactide-co-polycaprolactone films increased [1]. Raouche et al. found that the use of β-CD to encapsulate AITC proved to be efficient to protect the AITC against thermal degradation during processing [2]. It is possible for AITC encapsulated in CDs to be incorporated in antimicrobial food packaging systems. However, the effect of β-CD/AITC addition on the morphological, mechanical properties, and long-term controlled release characteristic of the PLA films has not been reported. Therefore, the aim of this research is to study the effect of different amounts of β-CD/AITC on morphological, mechanical properties, thermal characteristic of the PLA films, furthermore, their antimicrobial and controlled-release properties were also investigated.

## 2. Materials and Methods

### 2.1. Materials

Polylactic acid (PLA) 4032D, was obtained from Nature Works (New York, NY, USA); allyl isothiocyanate (≥99% purity) supplied by Xiya chemical Co., Ltd. (Chengdu, China); β-cyclodextrin (≥99% purity) was purchased from Sinopharm Chemical reagent Co., Ltd. (Shanghai, China) polyethylene glycol (PEG400) was purchased from Nature Works (New York, NY, USA); All of the strains, including bacteria (*Staphylocccus aureus*, *Escherichia coli*, *Salmonella*, *Bacillus subtitles*), and mould (*Aspergillus niger*, *Penicillium*) were provided by Jiangnan University (Wuxi, China). Other chemicals that were used were of analytical grade.

### 2.2. Preparation of the IC of β-CD/AITC

The IC of β-CD/AITC was prepared according to the method of Bhandari (1998), with some modifications [23]. Three percent (*w*/*v*) of β-CD were dissolved in 100 mL deionized water at 60 °C. Then, the resulting solutions were equilibrated in a water bath at 40 °C. Then, AITC in ethanol (1:1, *v*/*v*) was added drop-wise into the β-CD solutions to give a molar ratio of β-CD/AITC of 1.0, which were continually stirred using a magnetic stirrer for 3 h at a constant rate of 600 rpm. The suspensions were filtered, rinsed twice with absolute ethanol, and then freeze-dried to obtain the complex of β-CD/AITC. All of the supernatants were mixed together and the concentration of AITC was calculated with a UV spectrophotometer (248 nm) by using a standard curve plotted using different concentrations of AITC. The encapsulation efficiency was calculated using the following equation: Encapsulation efficiency (%) = (Total amount of AITC−Free AITC)/Total amount of AITC × 100.

The β-CD/AITC complex had a high encapsulation efficiency of 85.2%.

### 2.3. Preparation of Ternary Antimicrobial Films of β-CD/AITC/PLA

The antimicrobial films were prepared by a laboratory blown film extrusion line configured with a twin-screw extrusion system. The materials were blended in accordance with the formula shown in Table 1. The films were prepared by a two-step process. The first step is to obtain the polymer pellets by a twin-screw extrusion system. The temperature profile in each zone of the extruder was set as 100/130/157/172/174/163/160 °C from the polymer feeding to the die. After the extrusion, the polymer pellets were cooled and vacuum dried at 70 °C. In the second step, the films were prepared by blow-film extruder. The temperatures of the different zones of extruder were maintained at 130/150/160/170 °C, and the screw speed was maintained at 60 rpm.

### 2.4. Thickness of Films

The thickness of the films was determined using a digital micrometer (Vernier, China, 0.001 mm accuracy).

### 2.5. Mechanical Properties

The film’s tensile strength (TS), elongation at break (E), and elastic modulus (M) were measured at room temperature using Texture Analyzer (TA) (Lloyd Instruments, West Sussex, UK). The composite films were cut into strips (1 cm × 10 cm). The films were loaded into the testing system, which was set at an initial sample length and grip speed of 2 cm and 100 mm/min, respectively. TS were calculated by dividing maximum load by the film’s cross-sectional area. E% was expressed as the percentage of change in the original length of a specimen between the grips at break. M was the slope of the tensile stress–strain curve over the linear elastic region.

### 2.6. Differential Scanning Calorimetry (DSC)

The thermal properties of the film samples were investigated using a differential scanning calorimeter (DSC1, Mettler–Toledo, Schwerzenbach, Switzerland), according to the method reported by Chanvrier et al. with minor modifications [24]. Indium was used as the calibration standard. Each sample (approximately 5 mg) was placed in an aluminum pan and the container was hermetically sealed. The sample pans were heated from 30 °C to 200 °C at a rate of 10 °C/min to observe the presence of any residual enthalpy melting peaks.

### 2.7. Scanning Electron Microscopy (SEM)

The surfaces of the film samples were observed by a JSM-5610LV SEM (JEOL, Tokyo, Japan) with an acceleration voltage of 5 kV, according to the description of De la Caba et al. [25]. The films were frozen in liquid nitrogen, and then vacuum freeze-dried immediately. Prior to tophotographic observation, the fracture surfaces were sputter coated with a layer of gold to avoid charging under the electron beam.

### 2.8. Fourier Transform Infrared Spectroscopy Analysis

Fourier transform infrared (FTIR) spectra were recorded using a Nicolet 6700 spectrometer (Thermo Fisher Scientific Inc., Waltham, MA, USA). The samples were collected using the KBr pellet method. The spectra were obtained in transmittance mode at room temperature. The wavenumber range was from 4000 cm^−1^ to 400 cm^−1^. The resolution was 4 cm^−1^, and the total number of scan was 32.

### 2.9. Assay for Antimicrobial Activity

The antibacterial experiment was carried out by colony counting method, reported by Aytac et al., with some modifications [10]. The overnight culture of bacteria (*S. aureus*, *Salmonella*, *E. coli*, *B. subtilis*) and mold (*Penicillium*, *Aspergillus niger*) were adjusted to the final OD of 1.0 in a liquid medium. Samples from each flask were serially diluted, 100 μL of each was spread onto LB agar, simultaneously, about 1 mg of film was cut into cubic and put on the surface of agar, the culture dishes were incubated at 37 °C for 24 h. The growth inhibition rate (%) was calculated by the following formula:

Growth inhibition rate (%) = (Total colony number of blank sample − Total colony number of film samples)/Total colony number of blank sample × 100.

### 2.10. The Cumulative Release of AITC from Ternary Antimicrobial Films

The concentration of AITC in antimicrobial films was analyzed using headspace gas chromatography-mass spectrometry (GC-MS) of Agilent Technologies 7890A gas chromatograph coupled to an Agilent Technologies 5975C inert MSD (Walker Information, Inc., Beijing, China) with a triple-axis detector according to the method reported by Raouche et al. [2],. Helium was used as carrier gas was at a flow rate of 1.2 mL/min. 500 μL of vapor was injected to the headspace GC–MS by using a headspace injector. The oven temperature initially at 50 °C was raised by 4 °C/min to 150 °C, then by 15 °C/min to 250 °C, and was kept at 250 °C for 10 min. Injection was done in split mode with a ratio of 1:20.

The cumulative amounts of AITC released from ternary antimicrobial films were determined for 110 h by GC–MS according to the method described above. One gram of film samples were cut up and placed in 250 mL headspace glass vials. The vials were agitated at 500 rpm at 50%, 75%, 98% relative humidity (RH), controlled by saturated salt solutions at 40 °C. In addition, to investigate the influence of temperature, the samples were heated to 10, 25, 40 °C, respectively, and at 98% RH. The cumulative release was quantified as follows: Cumulative release (%) = (Released AITC/Total AITC in films) × 100.

### 2.11. Statistical Analysis

All of the experiments were conducted at least thrice, and the mean values and standard deviations were determined. The experimental data were analyzed using analysis of variance (ANOVA) and were expressed as mean values ± standard deviations. Differences were considered at a significance level of 95% (*p* < 0.05). Pearson’s correlation coefficients among the parameters were calculated using the Statistical Package for the Social Sciences (SPSS) version 17.0 software (SPSS Inc., Chicago, IL, USA).

## 3. Results and Discussion

### 3.1. Mechanical Properties of Antimicrobial Films

The tensile strength (TS), the elongation at break (E), and elastic modulus (M) of PLA films with different amounts of β-CD/AITC are presented in Table 2. PLA film showed an elastic modulus of 559 MPa with a tensile strength of 48.83 Mpa and an elongation at break of 61.33%, which was consistent with the results of Armentano et al. [22]. The addition of β-CD/AITC decreased the TS of the films, ranged from 48.83 to 30.51 Mpa. However, the E of PLA films increased significantly with the increase of β-CD/AITC. This could be due to the increase in the chain mobility of PLA composite films with β-CD/AITC. The composite film with 10% β-CD/AITC had the highest E of 88.56% (Table 2). However, when the percentage of an additional β-CD/AITC was 15–20%, the E of the composite films decreased. This phenomenon was probably due to the agglomeration of β-CD/AITC, leading to micro-phase separation. The M of PLA films decreased significantly with an increase of β-CD/AITC, and ranged from 559 Mpa to 327 Mpa. Similar results were reported by Armentano et al. [22] for PLA films with carvacrol, in which the M of composite films decreased. They suggested that carvacrol could act as plasticizer agents, increasing the chain mobility of the macromolecules. Films used in food packaging require flexibility to avoid breaking during the packaging procedure, while a minimum value of hardness is also a requirement to avoid perforations during their transport and exposition lifecycle.

### 3.2. Thermal Properties of Antimicrobial Films

**Figure 1:**
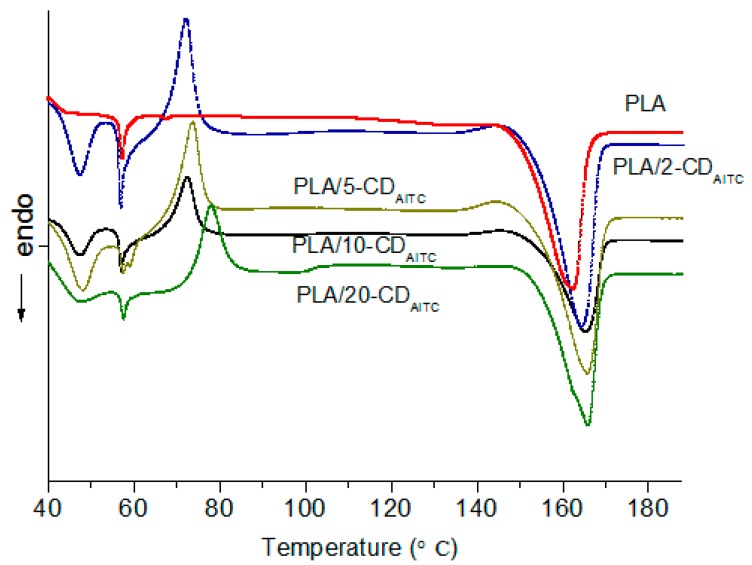
Differential Scanning Calorimetry (DSC) thermograms of the PLA film with different amounts of β-CD/AITC. PLA/2-CD_AITC_, PLA/5-CD_AITC_, PLA/10-CD_AITC_, PLA/20-CD_AITC_: PLA films with 2%, 5%, 10%, 20% β-CD/AITC, respectively.

The melting onset, peak, and conclusion temperature (To, Tp, and Tc, respectively) and melting enthalpy (ΔH) of the PLA films with different amounts of β-CD/AITC are presented in Figure 1, and the thermal parameters are summarized in Table 3. The melting temperature range of PLA film was 143.61–170.34 °C, and the ΔH was 27.58 J/g. When compared to PLA film, the melting temperature of the PLA film with different amounts of β-CD/AITC increased significantly, and the PLA film with 10% β-CD/AITC showed the highest Tp (166.03 °C). The addition of β-CD/AITC increased significantly ΔH of the PLA films, thus indicating a more orderly crystal structure. The higher melting temperature and ΔH of PLA films with the β-CD/AITC may be attributed to the interactions between PLA and β-CD in the process of extrusion. Similarly, Plackett et al. reported that the incorporation of the CD complexes in the polylactide-co-polycaprolactone films enhanced the melting temperature and ΔH of the films [1].

### 3.3. SEM Images of Antimicrobial Films

**Figure 2:**
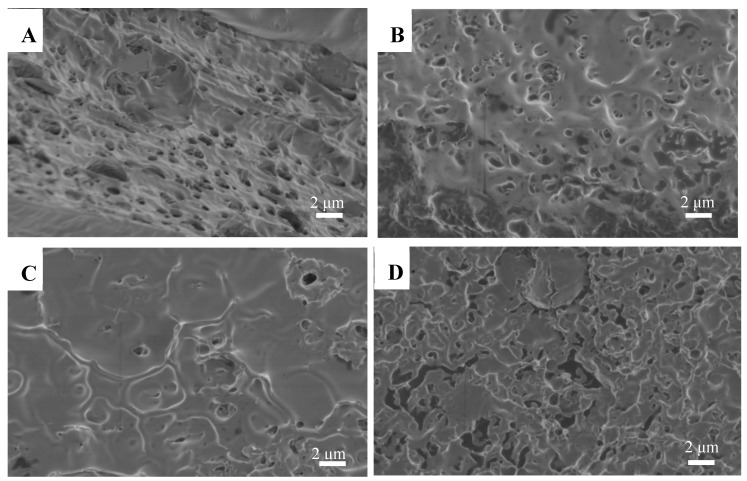
SEM images of the surface of the antimicrobial PLA films with different amounts of β-CD/AITC ((**A**) PLA film; (**B**–**D**) PLA films with 2%, 10%, 20% β-CD/AITC, respectively).

The surface morphology of the antimicrobial PLA films with different amounts of β-CD/AITC is presented in Figure 2. The control PLA films had a relatively rough surface with many small holes (Figure 2A). The incorporation of β-CD/AITC into the films promoted changes in the film's morphology. With the incorporation of β-CD/AITC into the PLA films, the microstructure became compact and no phase separation occurred, which could be due to the formation of hydrogen-bonding interactions between PLA and β-CD/AITC. However, with 20% β-CD/AITC, the films became rough and many small pores could be observed. This could be due to uneven dispersion and local enrichment of β-CD/AITC in PLA films. This is believed to be caused by strong intermolecular interactions leading to improved mechanical properties (Table 1) and thermal stability (Table 2) of the PLA films.

### 3.4. Fourier Transformation Infrared Spectroscopy Analysis

**Figure 3:**
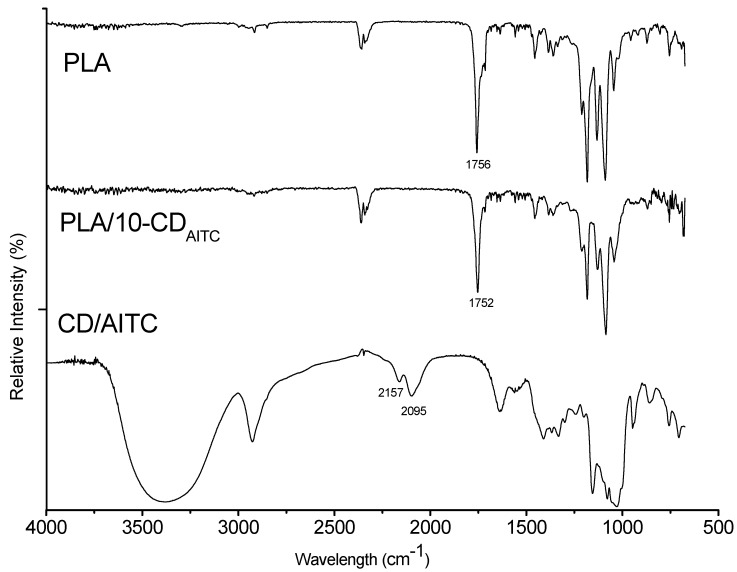
Fourier transformation infrared spectroscopy curves of the β-CD/AITC and PLA film with and without 10% β-CD/AITC.

To further investigate the interaction between β-CD/AITC and PLA, FITR spectra of β-CD/AITC and PLA film with and without 10% β-CD/AITC were determined, and the results are presented in Figure 3. For the β-CD/AITC, the strong absorption band in the range of 3700–3000 cm^−1^ was attributed to the O–H stretching of the β-CD. The band at about 2926 cm^−1^ was characteristic of C–H stretches associated with ring methane hydrogen atoms of β-CD. However, the bands at 2157 cm^−1^ and 2095 cm^−1^ were a feature peak of −N=C=S in AITC, which occur strongly because of fermi resonance. This result indicated that AITC successfully formed a complex with β-CD. For the PLA film, the band at 1756 cm^−1^ was due to the stretching vibration of carbonyl of PLA [24]. In composite films (PLA/10-CD_AITC_), this peak was shifted towards a lower wave number (1752 cm^−1^), which might be due to the hydrogen bonding interactions between the carbonyl of PLA and hydroxyl groups of β-CD/AITC. Similarly, Cano et al., reported that the bands shifted to a shorter wavelength owing to the hydrogen bonding interactions between pea starch and poly vinyl alcohol chains [26].

### 3.5. Antimicrobial Activity

**Figure 4:**
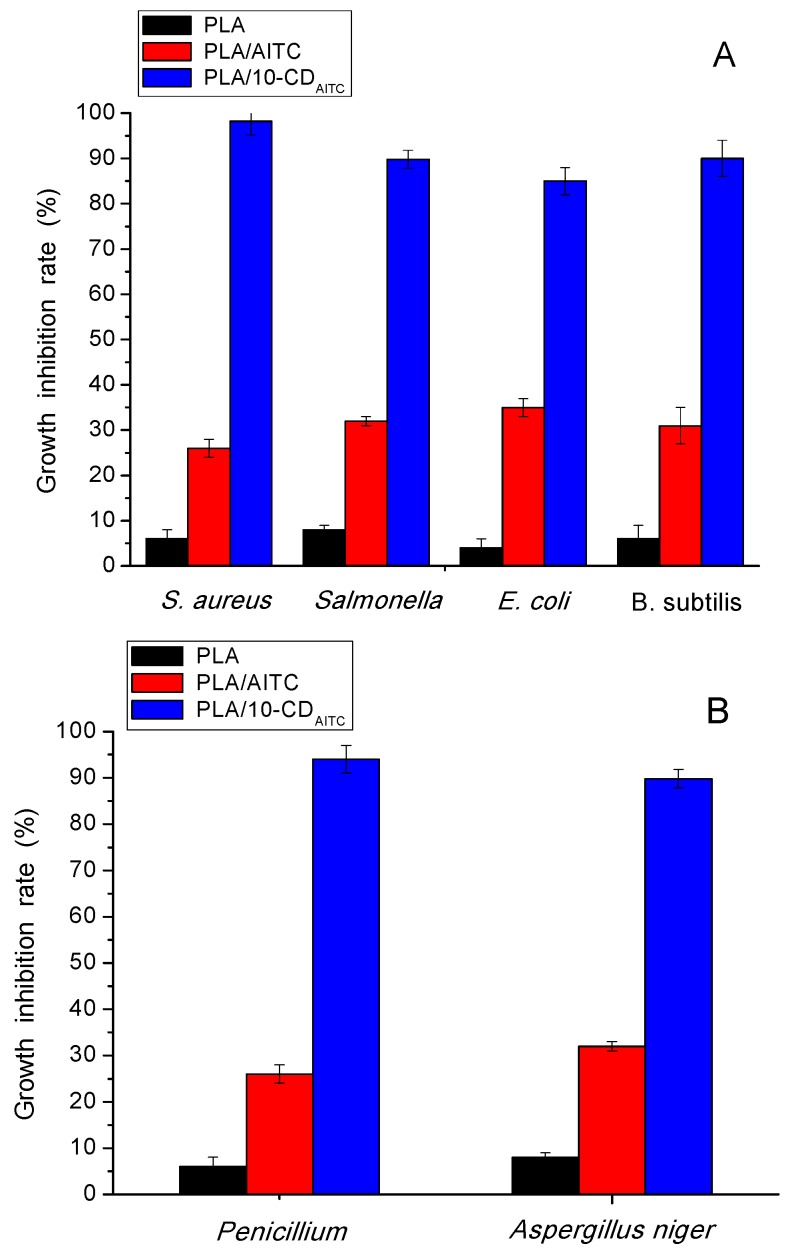
Growth inhibition rate (%) of bacteria (**A**) (*S. aureus*, *Salmonella*, *E. coli*, *B. subtilis*) and mold (**B**) (*Penicillium*, *Aspergillus niger*) in PLA film, PLA film with 10% β-CD/AITC, and PLA film with allyl isothiocyanate (AITC).

The antimicrobial activity of PLA film with and without 10% β-CD/AITC and PLA film with AITC is shown in Figure 4. PLA film exhibited almost no antimicrobial activity against bacteria (*S. aureus*, *Salmonella*, *E. coli*, *B. subtilis*) and mold (*Penicillium*, *Aspergillus niger*). The PLA film with AITC had an inhibition ratio of 26–35%. The lower growth inhibition ratio was mainly due to the thermal degradation of AITC during the extrusion process. An obvious increase in antimicrobial activity of PLA film with β-CD/AITC was observed. This could be because the use of β-CD provided effective protection for AITC against thermal degradation during processing [1]. The PLA film with 10% β-CD/AITC showed better activity against gram-positive (*S. aureus*, *Salmonella*, *B. subtilis*) than against gram-negative (*E. coli*). Similar findings were previously reported by Aytac et al. [10], who found that β-CD/AITC incorporated in polyvinyl alcohol (PVA) nanofibers were more active against gram-positive than against gram-negative bacteria. In addition, the composite films with β-CD/AITC exhibited a high growth inhibition ratio against bacteria (85–98%) and mold (88–94%), which suggested that the composite films with β-CD/AITC had a broad spectrum of antibacterial activities.

### 3.6. Release Kinetics Study

**Figure 5:**
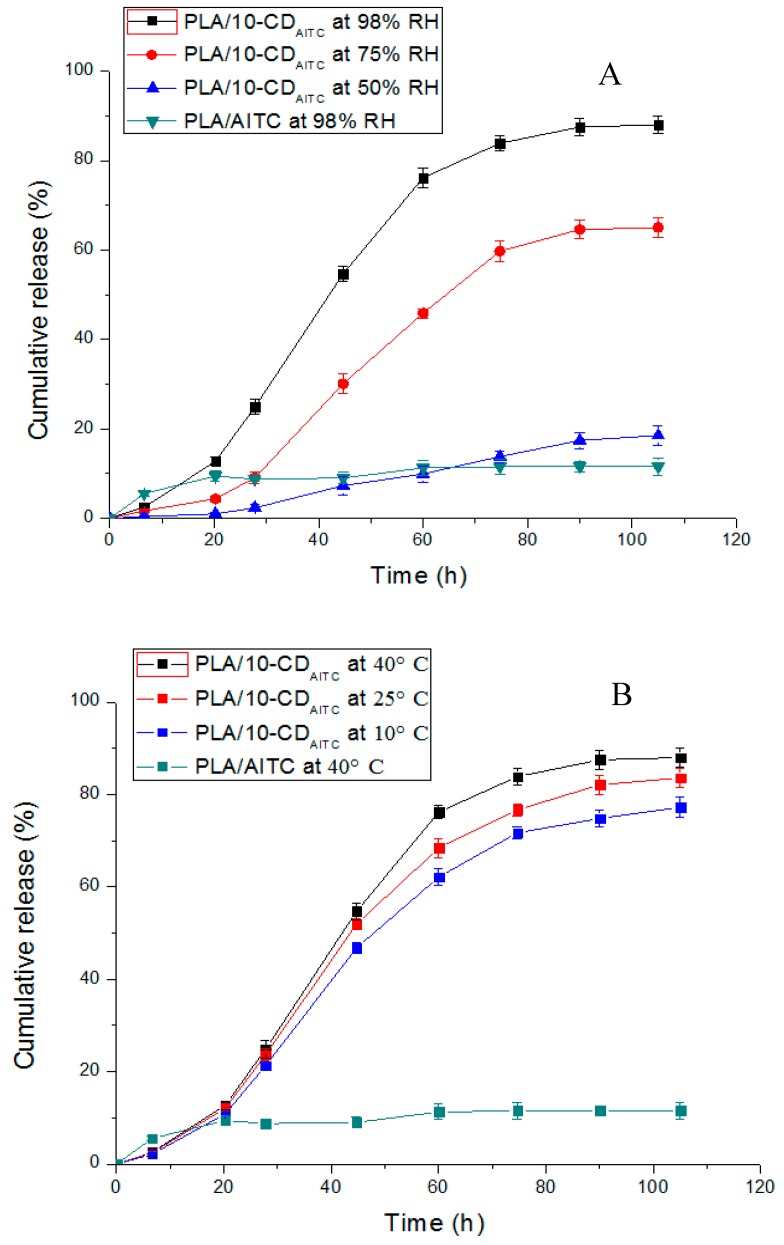
Release behavior of AITC from AITC/PLA and β-CD/AITC/PLA films, respectively, at different relative humidity (RH) ((**A**), at 40 °C) and temperature ((**B**), at 98% RH).

The release kinetics of AITC from AITC/PLA film and β-CD/AITC/PLA film, respectively, at different RH and temperature were investigated, and the results obtained, as shown in Figure 5. The release of AITC from AITC/PLA film was fast during the first 7 h, but only about 10% of AITC from was finally released indicating that most of AITC was degraded during extrusion. However, the release of AITC from film of β-CD/AITC/PLA was slower and more sustained at different RH and temperature (Figure 5A,B). Furthermore, a moisture-activated release mechanism was found for β-CD/AITC/PLA film [27]. The triggered release RH was 75%. With the increase in time, AITC could be gradually released from the film, and its release rate was greatly accelerated, followed by a slight increase until 110 h (Figure 5A). In addition, the higher AITC release was observed at 98% RH than that for 75% RH. The presence of water was essential for the formation of the IC between CDs and hydrophobic guests. The inclusion reaction was a substitution process of guest for water in the cavity of the CD and vice versa [28]. Therefore, the release of AITC from β-CD might be markedly affected by the environmental RH. These results indicated that the storage condition of the antimicrobial package made by β-CD/AITC/PLA film should avoid high moisture.

To evaluate the effect of temperature on the release of AITC, those two films were placed at different temperatures (10 °C, 25 °C, and 40 °C, respectively), and the cumulative release results of AITC are shown in Figure 5B. At 40 °C, AITC released more and faster than that at 10 and 25 °C, with approximately 90% release from the β-CD/AITC/PLA films. These results demonstrated that the release of AITC were dependent on both temperature and RH.

## 4. Conclusions

The ternary antimicrobial films of β-CD/AITC/PLA were successfully prepared by two-step extrusion method. The elongation of the composite films increased from 61.33% to 88.56%, indicating that β-CD/AITC could be used to greatly improve film flexibility. The addition of β-CD/AITC significantly increased the thermal stability of PLA films. The DSC result showed that the Tp of ternary composite films increased significantly, and the PLA films with 10% β-CD/AITC showed the highest Tp (166.03 °C), suggesting that the compact structure between β-CD/AITC and PLA was formed, which can be confirmed by SEM image of ternary composite films. The FTIR results revealed that the interactions between β-CD/AITC and PLA films occurred. The controlled release behavior of AITC encapsulated in β-CD was significantly affected by relative humidity and temperature. The ternary antimicrobial films of β-CD/AITC/PLA can be applied as an effective antimicrobial packing material for food and other industries.

## Figures and Tables

**Table 1:** Composition and labels of antimicrobial films.

Label	Composition	Added Active Compound Content (wt. %)
PLA	90% PLA + 10% PEG	0
PLA/AITC	92% (PLA:PEG = 9:1) + 8% AITC	8%
PLA/2-CD_AITC_	98% (PLA:PEG = 9:1) + 2% β-CD_AITC_	0.16%
PLA/5-CD_AITC_	95% (PLA:PEG = 9:1) + 5% β-CD_AITC_	0.40%
PLA/10-CD_AITC_	90% (PLA:PEG = 9:1) + 10% β-CD_AITC_	0.80%
PLA/15-CD_AITC_	85% (PLA:PEG = 9:1) + 15% β-CD_AITC_	1.20%
PLA/20-CD_AITC_	80% (PLA:PEG = 9:1) + 20% β-CD_AITC_	1.60%

Polylactic acid (PLA) represents PLA film. PLA/2-CD_AITC_, PLA/5-CD_AITC_, PLA/10-CD_AITC_, PLA/15-CD_AITC_, PLA/20-CD_AITC_ represents PLA films containing 2%, 5%, 10%, 15%, 20% β-CD/AITC respectively.

**Table 2:** Mechanical properties of PLA films with different amounts of β-CD/AITC.

Label	Thickness (μm)	TS (MPa)	E (%)	M (Mpa)
PLA	26.59 ± 0.23 ^e^	48.83 ± 0.99 ^a^	61.33 ± 0.86 ^e^	559 ± 23 ^a^
PLA/2-CD_AITC_	27.22 ± 0.41 ^e^	46.62 ± 0.82 ^b^	76.77 ± 0.68 ^c^	503 ± 36 ^b^
PLA/5-CD_AITC_	28.42 ± 0.62 ^d^	42.64 ± 1.03 ^c^	80.26 ± 1.12 ^b^	475 ± 28 ^c^
PLA/10-CD_AITC_	32.16 ± 0.68 ^c^	39.79 ± 1.21 ^d^	88.56 ± 1.26 ^a^	380 ± 35 ^d^
PLA/15-CD_AITC_	35.21 ± 0.87 ^b^	33.56 ± 0.83 ^e^	65.31 ± 0.97 ^d^	385 ± 31 ^d^
PLA/20-CD_AITC_	38.64 ± 1.12 ^a^	30.51 ± 0.96 ^f^	56.79 ± 0.75 ^f^	327 ± 26 ^e^

Values represent the mean ± standard deviation of triplicate tests. Values in the same column having different letters were significantly different (*p* < 0.05). TS represents tensile strength, E represents elongation at break, and M represents elastic modulus. PLA represents PLA film. PLA/2-CD_AITC_, PLA/5-CD_AITC_, PLA/10-CD_AITC_, PLA/15-CD_AITC_, PLA/20-CD_AITC_ represents PLA film containing 2%, 5%, 10%, 15%, 20% β-CD/AITC respectively.

**Table 3:** Thermal properties of the PLA films with differen t amounts of β-CD/AITC.

Sample	To (°C)	Tp (°C)	Tc (°C)	ΔH (J·g ^−1^)
PLA	143.61 ± 1.22 ^d^	161.62 ± 0.64 ^d^	170.34 ± 0.62 ^c^	27.58 ± 0.62 ^d^
PLA/2-CD_AITC_	144.12 ± 1.35 ^c^	164.80 ± 0.47 ^c^	171.26 ± 0.53 ^b^	38.23 ± 0.46 ^b^
PLA/5-CD_AITC_	144.63 ± 1.26 ^bc^	165.17 ± 0.63 ^b^	172.41 ± 1.21 ^a^	40.46 ± 0.42 ^a^
PLA/10-CD_AITC_	146.31 ± 0.93 ^b^	166.03 ± 0.37 ^a^	172.11 ± 1.17 ^a^	39.51 ± 0.73 ^a^
PLA/20-CD_AITC_	148.16 ± 0.83 ^a^	165.40 ± 0.28 ^b^	170.23 ± 0.89 ^c^	35.48 ± 0.25 ^c^

Values represent the mean ± standard deviation of triplicate tests. Values in the same column having different letters were significantly different (*p* < 0.05). PLA represents PLA film. PLA/2-CDAITC, PLA/5-CDAITC, PLA/10-CDAITC, PLA/20-CDAITC represents PLA film containing 2%, 5%, 10%, 20% β-CD/AITC respectively.
